# Supplementary material for: A fungal extracellular effector inactivates plant polygalacturonase-inhibiting protein
Source: Nat Commun. 2022 Apr 25;13:2213. doi: 10.1038/s41467-022-29788-2 (PMC9038911; doi:10.1038/s41467-022-29788-2)
Supplement: Supplementary file 1 — Supplementary Information [file 41467_2022_29788_MOESM1_ESM.pdf]

Supplementary Information

A novel fungal extracellular effector inactivates plant polygalacturonase-inhibiting protein

Wei Wei et al.

Supplementary Figures 1 to 11

Supplementary Tables 1 to 4

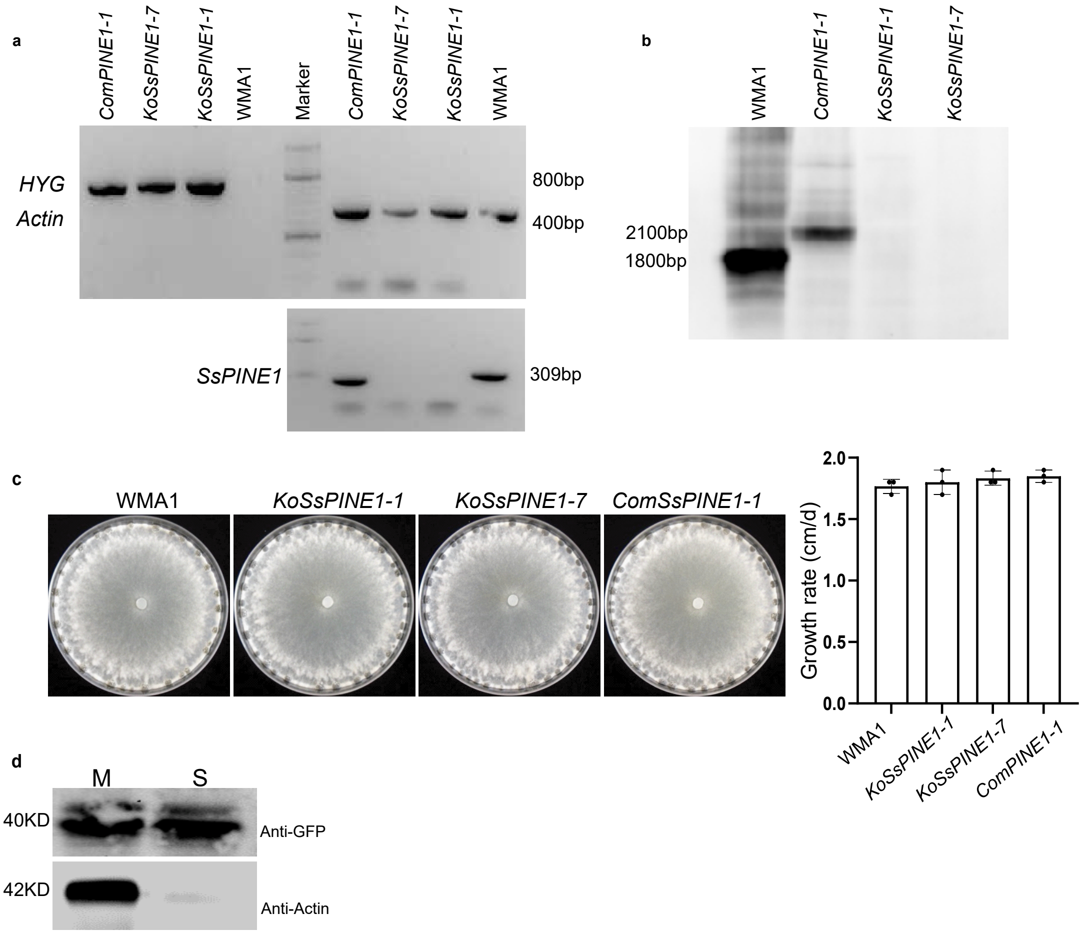

Supplementary Fig. 1. Characterization of *SsPINE1*-deletion mutants. a Detection of *SsPINE1* and *hygromycin B phosphotranferase* (*HYG*) transcripts in wildtype WMA1, SsPINE1-deletion mutants *KoSsPINE1-1* and *KoSsPINE1-7*, and SsPINE1-complemented strain *ComSsPINE1-1* by RT-PCR. The *Actin* gene was used as a positive control. **b** Confirmation of *SsPINE1* deletion by Southern Blot using *SsPINE1* as a probe. **c** Colony morphology (7 day old culture) and growth rate of the first two days of WMA1, *KoSsPINE1-1* and *KoSsPINE1-7*, and *ComSsPINE1-1* on the PDA medium. Data represent mean ± s.d. (n = 3 plates). No difference in growth rate was observed. The experiment was performed three times independently with similar results obtained. **d** SsPINE1 is a secreted protein. SsPINE1-GFP was detected mycelium extract (M) and in culture filtrate (S) of *ComSsPINE1-1* using anti-GFP antibody, whereas *Actin* can be detected only in mycelium extract.

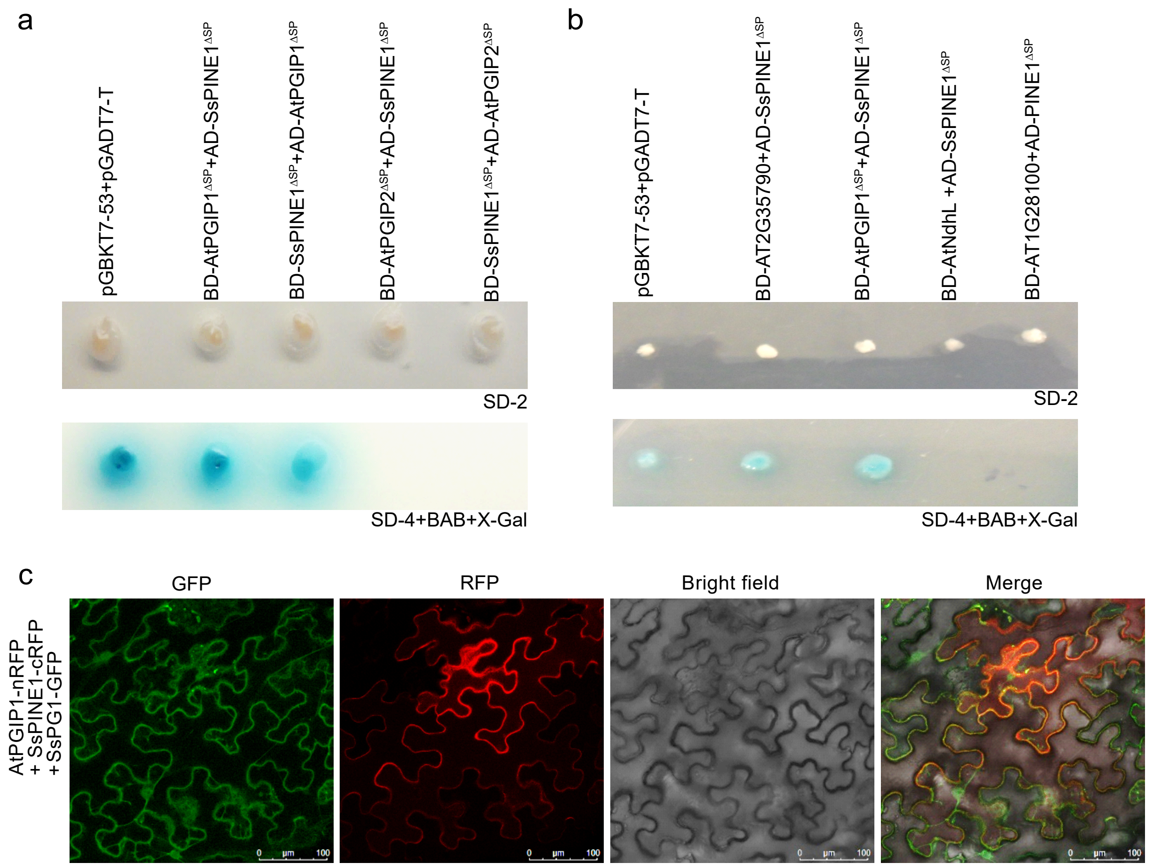

Supplementary Fig. 2. SsPINE1 interaction with potential prey proteins and its co-localization with AtPGIP1 and SsPG1. **a** Yeast two-hybrid assay showed that SsPINE1 interacted with AtPGIP1 but not with the other gene family member AtPGIP2, as either the bait or the prey. **b** Yeast two-hybrid assay showed SsPINE1 interacted with the mitochondrial transmembrane protein At2G35790 and AtPGIP1 but not with AtNdhL (At2G1110) or At2G28100. **c** Co-localization of three proteins AtPGIP1, SsPG1 and SsPINE1. *N. benthamiana* leaves were co-infiltrated with a mixture of *Agrobacterium tumefaciens* strains harboring AtPGIP1-nRFP, SsPINE1-cRFP and SsPG1-GFP constructs. Fluorescence signals were monitored at 2 days post-agroinfiltration using confocal laser scanning microscopy. Representative images are shown. The experiment was performed three times with similar results obtained.

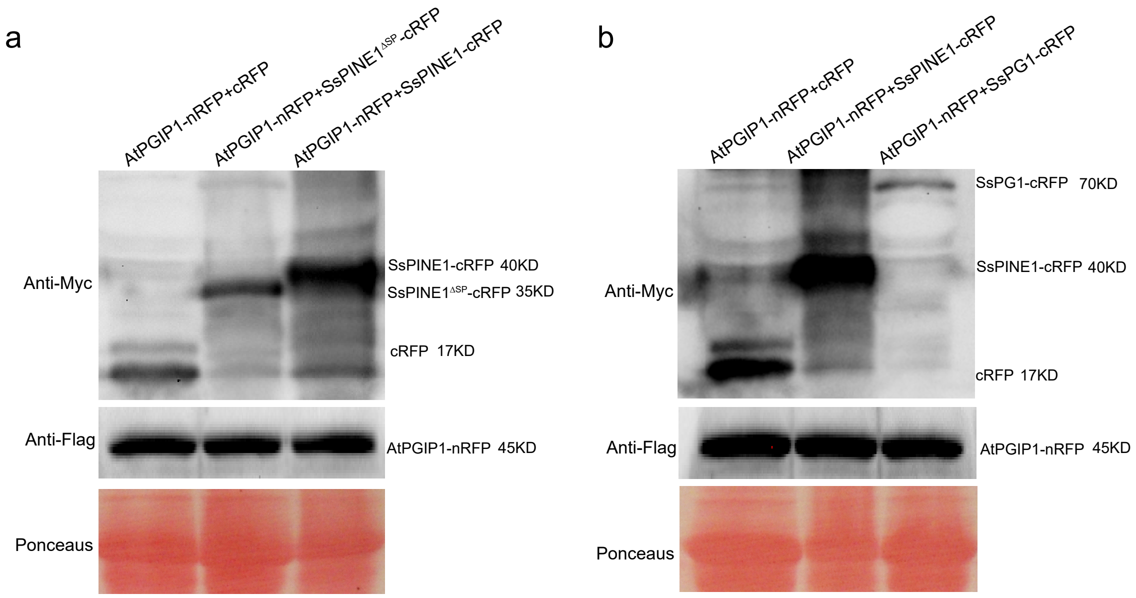

Supplementary Fig. 3. Verification of construct expression during bimolecular fluorescence complementation (BiFC). a Western blots showed that BiFC construct-encoded proteins were all expressed in *N. benthamiana* leaves used in Fig. 1c. The vector for cRFP carries a Myc tag. **b** Western blot detection of BiFC construct-encoded proteins expressed in *N. benthamiana* leaves used in Fig. 2b.

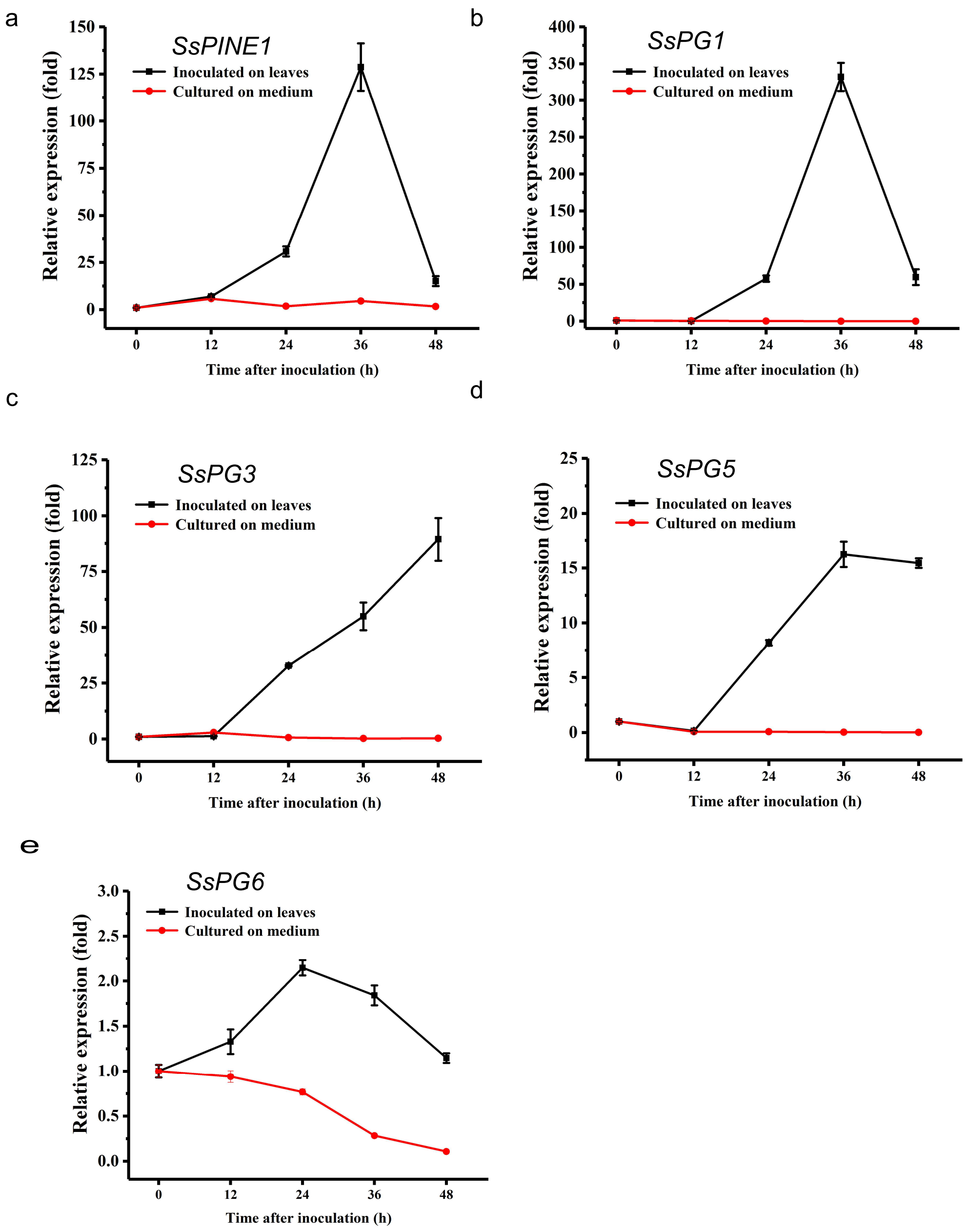

Supplementary Fig. 4. Comparison of expression patterns of *S. sclerotiorum PG* genes with that of *SsPINE1*. Gene expression analysis of *SsPINE1, SsPG1, SsPG3, SsPG5*, and *SsPG6* of wildtype strain WMA1 during plant infection and on PDA via RT-qPCR. The relative expression of *SsPINE1* and *SsPG1* are both significantly up-regulated 36 h after inoculation of *Arabidopsis thaliana* (Col-0) leaves. Their expression levels at 0 hpi were set as 1.0. *β-tubulin* expression levels were used for sample normalization. Data represent means ± s.d. (n = 3 either inoculated leaves or plates). The experiments were each performed twice with similar results obtained.

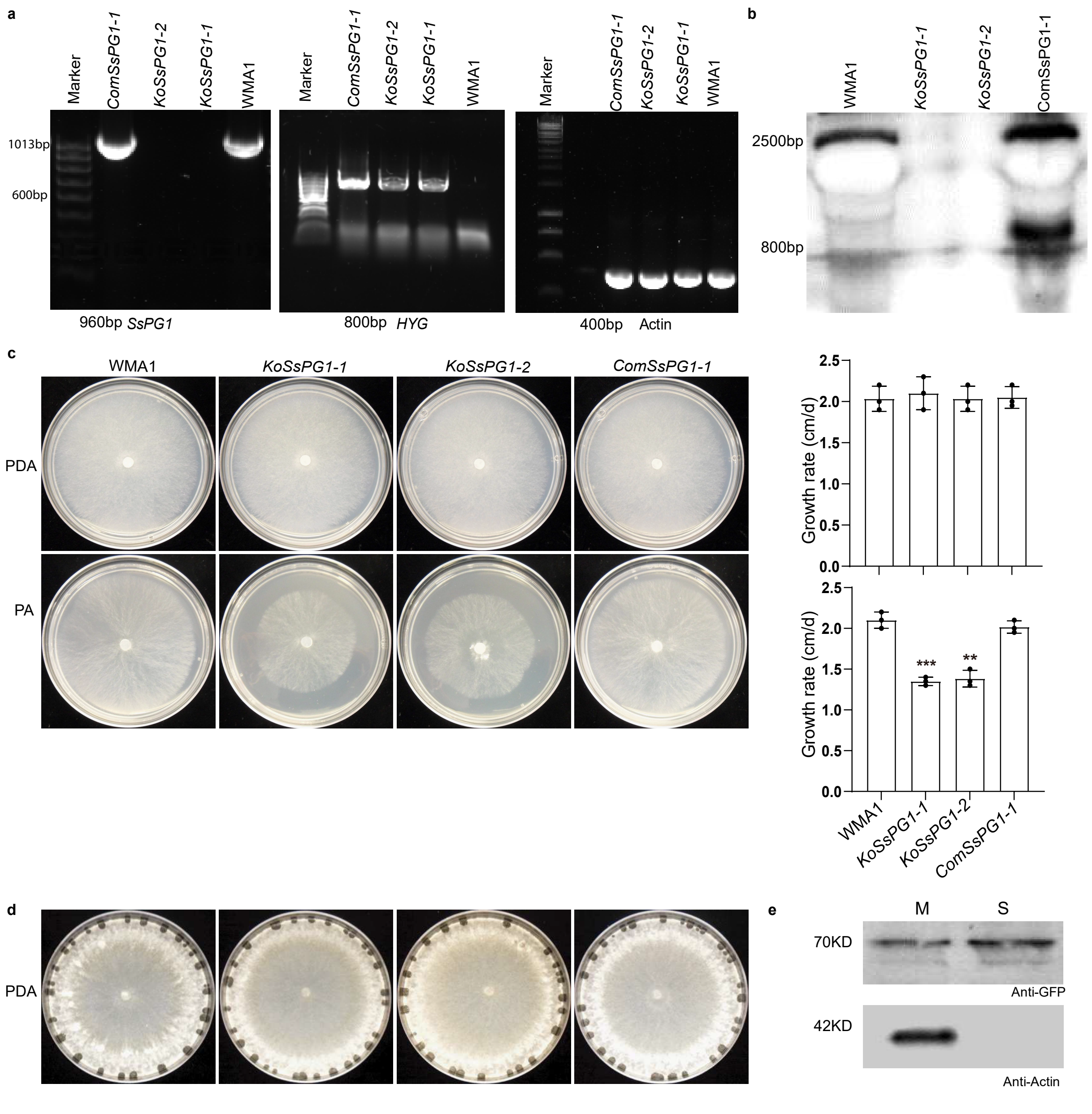

Supplementary Fig. 5. Characterization of *SsPG1*-deletion mutants. a Detection of *SsPG1* and *HYG* transcripts in wildtype strain WMA1, *SsPG1*-deletion mutants *KoSsPG1-1* and *KoSsPG1-2*, and *SsPG1*-complemented strain *ComSsPG1-1* by RT-PCR. *Actin* was used as a positive control. **b** Confirmation of *SsPG1*-deletion by Southern blot analysis using *SsPG1* as a probe. **c** Colony morphology and growth rate of WMA1, *KoSsPG1-1* and *KoSsPG1-2*, and *ComSsPG1-1* on potato dextrose agar (PDA) and 2% polygalacturonic acid medium (PA) at 2

dpi. Data represent mean ± s.d. (n = 3 plates). ** and *** indicate significant difference from the wildtype WMA1 at p<0.01 and 0.001, respectively, in two-tailed t test. The experiment was performed three times independently with similar results obtained. **d** Colony morphology (sclerotial formation) on PDA 10 dpi. The experiment was performed three times independently with similar results obtained. **e** SsPG1 is a secreted protein. SsPG1-GFP was detected in mycelium extract (M) and culture filtrate (S) of *ComSsPG1-1* using anti-GFP antibody, whereas Actin can be detected only in mycelium extract.

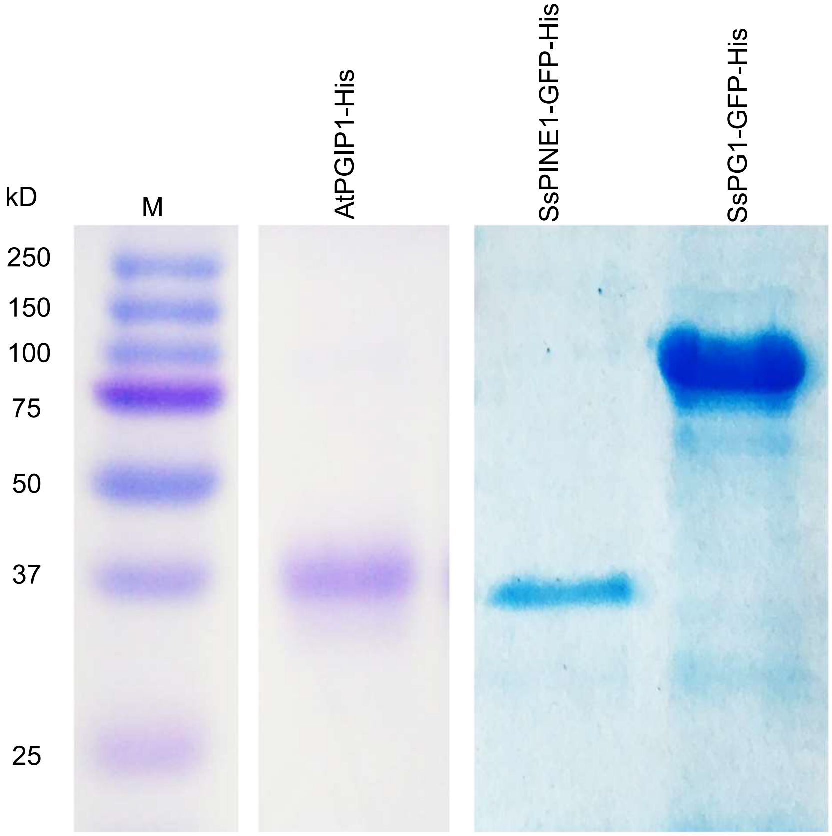

Supplementary Fig. 6. Purification of AtPGIP1, SsPINE1-GFP, and SsPG1-GFP proteins. SDS Polyacrylamide gel electrophoresis (SDS-PAGE) showed AtPGIP1-His, SsPINE1-GFP-His and SsPG1-GFP-His proteins purified from heterogenous expression in yeast.

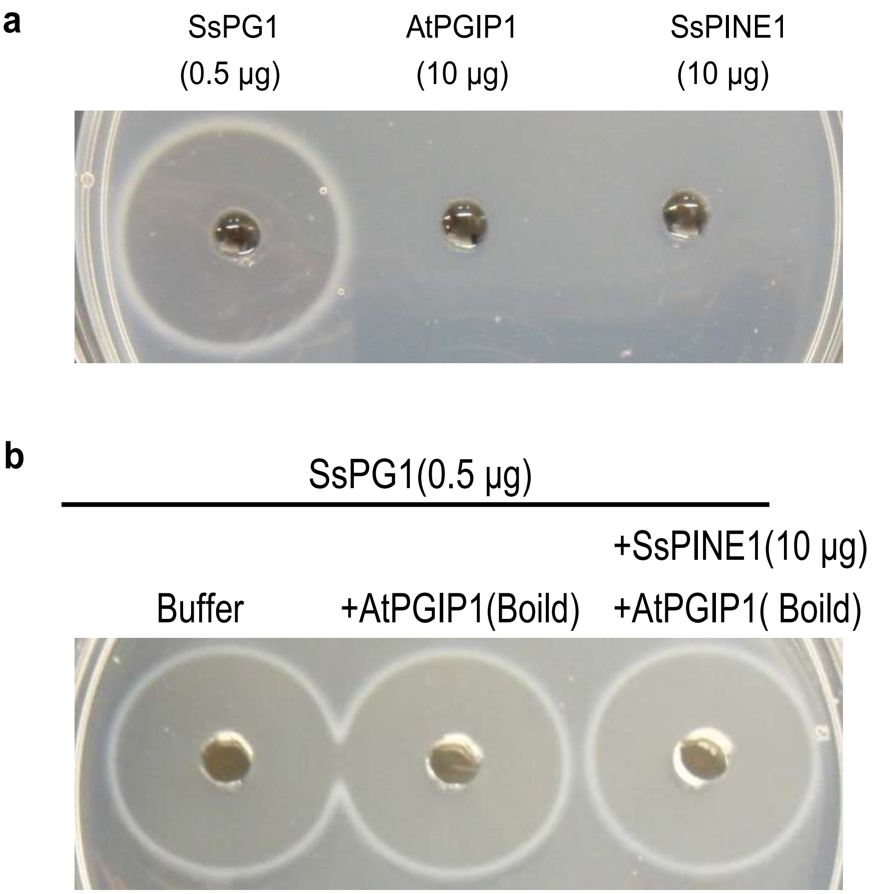

Supplementary Fig. 7. Agarose diffusion assays for polygalacturonase and inhibitor activities. **a** Purified protein was applied to 5-mm wells in 0.8% agarose gel containing 0.5% polygalacturonic acid. The halo rings indicate PG enzyme activity. Only SsPG1 exhibited polygalacturonase activity, whereas neither AtPGIP1 nor SsPINE1 showed any detectable PG activity. **b** Boiled AtPGIP1 protein lost its inhibition on PG activity of SsPG1.

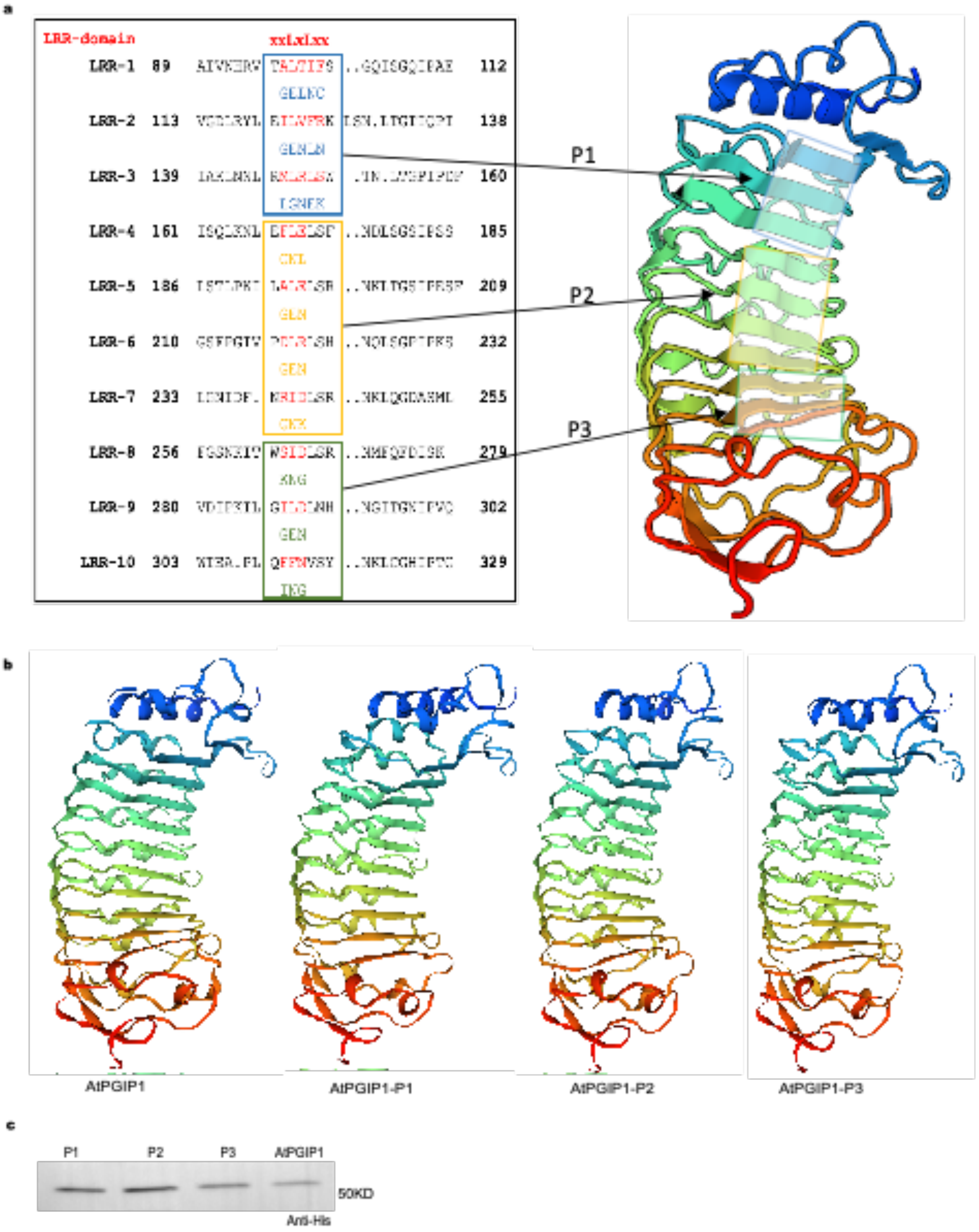

Supplementary Fig. 8 Mutagenesis of AtPGIP1 generates three mutation variants targeting P1, P2 or P3 motif. a Schematic diagram illustrating amino acid substitutions in three regions (P1, P2 and P3) of AtPGIP1 β-sheet-formed concave surface. The corresponding locations of the

substitutions in AtPGIP1 3D structure were also shown. The amino acid residues in red were substituted with the amino acid residues indicated below. **b** Three-dimensional (3D) structures of AtPGIP1, AtPGIP1-P1, AtPGIP1-P2 and AtPGIP1-P3. The mutations did not change the secondary structure of AtPGIP1 in modeling simulations. **c** Immunoblot analysis of the mutation variant and the native AtPGIP1 proteins purified form *Pichia pastoris*.

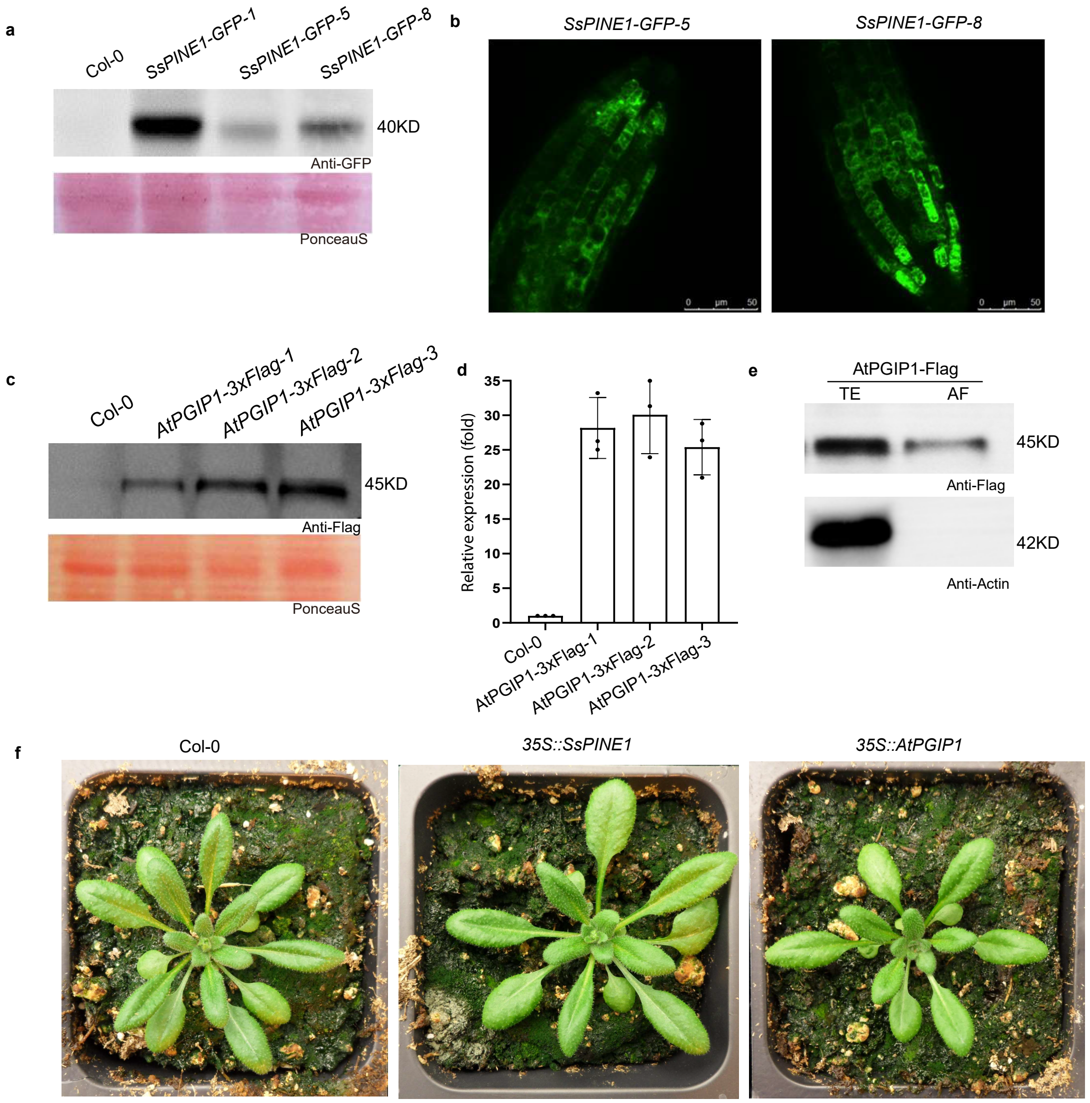

Supplementary Fig. 9. Verification and characterization of transgenic *Arabidopsis* plants expressing *SsPINE1-GFP* and *AtPGIP1-3xFlag*. **a** Expression of *SsPINE1-GFP* in transgenic lines was detected by Western blot using anti-GFP antibody. **b** Expression of *SsPINE1-GFP* in transgenic lines was detected via fluorescent microscopy. Roots of two-week-old plants were examined using a confocal microscope. **c** Expression of *AtPGIP1-3xFlag* in transgenic lines was detected by Western blot using an anti-Flag antibody. **d** Expression of *AtPGIP1-3xFlag* in transgenic lines was determined by RT-qPCR. Expression levels were normalized using *UBQ5* gene expression as the reference. Data represent mean ± s.d. (n = 3 plants). **e** AtPGIP1 is a secreted protein, which was detected in both leaf tissue (TE) and apoplastic fluid (AF) of *AtPGIP1-3xFlag*-expressing plant by Western blot using anti-Flag antibody. **f** Transgenic *A.*

thaliana (Col-0) lines expressing *SsPINE1-GFP* and *AtPGIP1-3xFlag* show growth rate and morphology similar to that of the wild type. Representative four-week-old plants were photographed four weeks after planting.

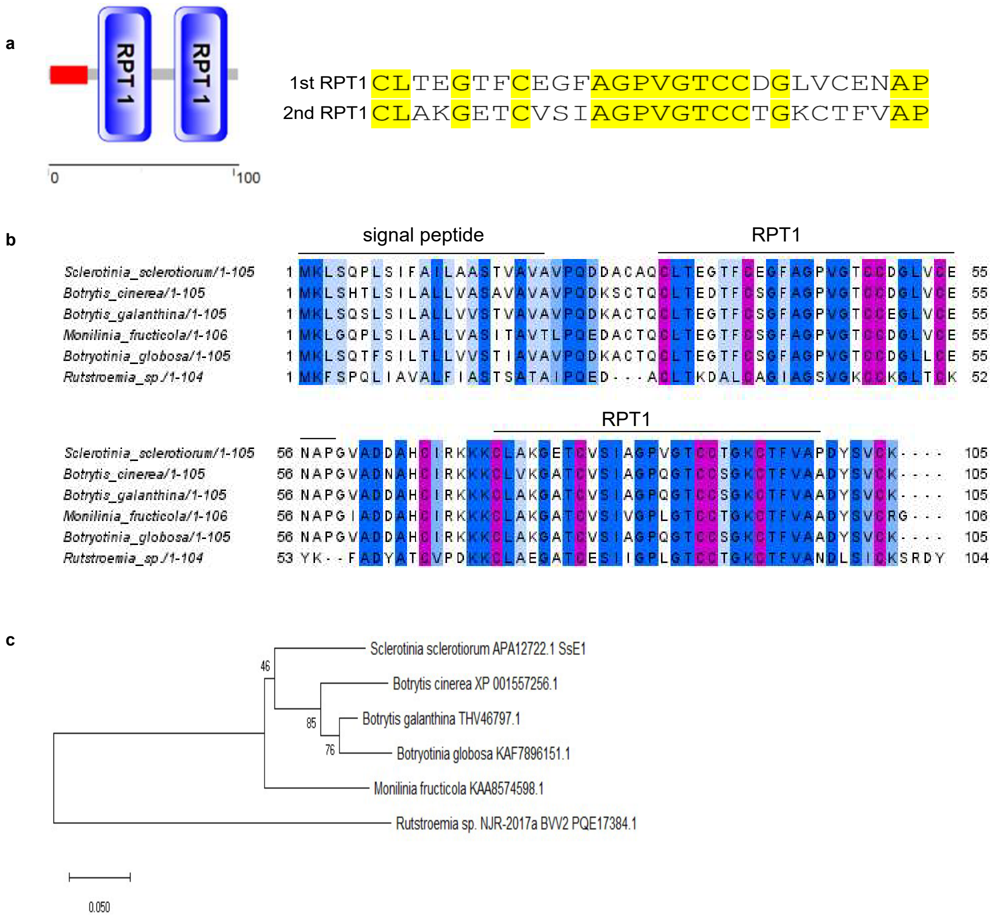

Supplementary Fig. 10. SsPINE1 structure and its relationship with its homologues. a Schematic representation of the signal peptide (red bar) and two internal repeats (RPT1) of SsPINE1 detected using the SMART model (http://smart.embl-heidelberg.de/smart/show_motifs.pl). The two internal repeats are shown in alignment and yellow shading indicates identical residues. **b** Multiple sequence alignment among SsPINE1 and its homologues found in other necrotrophic pathogens. Blue shading reflects levels of amino acid identify at each position. The twelve conserved cysteine residues are indicated in red. GenBank accessions of the proteins are indicated in **c**. **c** Phylogenetic tree showing the relationship of

SsPINE1 with the other homologues, analyzed using MEGA 5 with Unrooted neighbor-joining method using boot strap (1000 replications). The scale bar represents genetic distance of 0.05.

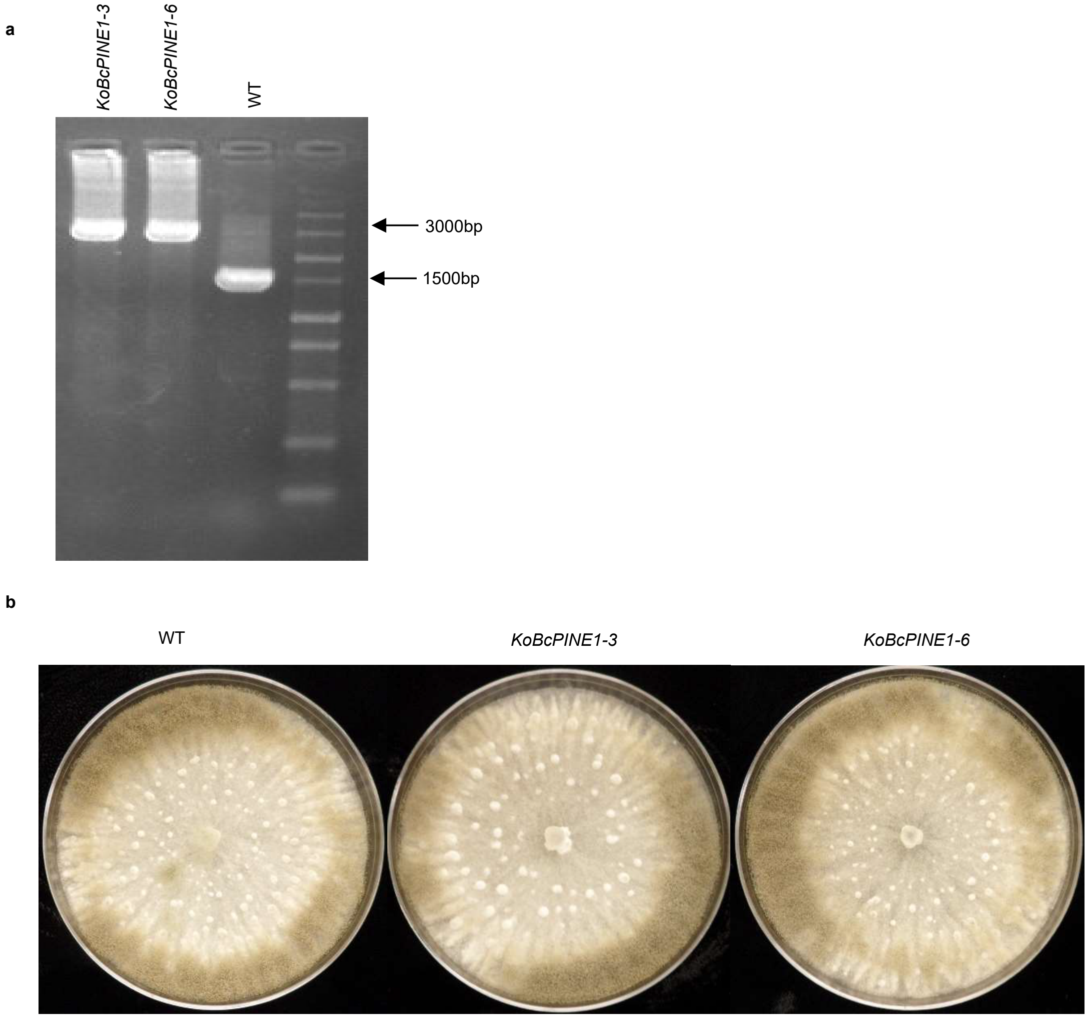

Supplementary Fig. 11. Characterization of *BcPINE1*-deletion mutants of *Botrytis cinerea.*
a. PCR amplification of the *BcPINE1* locus, showing the expected wildtype size in wildtype strain B05.10 and the increased size due to replacement by the hygromycin resistance cassette in the *BcPINE1* knockout mutants (KoBcPINE1-3 and KoBcPINE1-6) using PCR primers flanking the locus. **b** Colony morphology of *Botrytis cinerea* wildtype B05.10 and *BcPINE1*-deletion mutants KoBcPINE1-3 and KoBcPINE1-6 strains on PDA under continuous light 10 dpi, showing no observable differences in growth rate and colony morphology.

Supplementary Table 1. *Arabidopsis thaliana* prey proteins that interacted with SsPINE1 in two independent yeast two-hybrid screens (the top five prey proteins showed up in both Y2H screens)

GeneID	Function	# Colonies
NM_129137.4	Arabidopsis thaliana transmembrane protein (AT2G35790), mRNA	7
NM_120769.2	Arabidopsis thaliana polygalacturonase inhibiting protein 1 (PGIP1), mRNA	4
NM_105744.4	Arabidopsis thaliana inorganic carbon transport protein-like protein (NdhL), mRNA	4
NM_126278.3	Arabidopsis thaliana tobamovirus multiplication protein 3 (TOM3), mRNA	4
NM_102575.3	Arabidopsis thaliana hypothetical protein (AT1G28100), mRNA	3
NM_105441.3	Arabidopsis thaliana beta-ketoacyl reductase 1 (KCR1), mRNA	2
NM_129039.4	Arabidopsis thaliana DnaJ/Hsp40 cysteine-rich domain superfamily protein (EDA3), mRNA	2
NM_106024.6	Arabidopsis thaliana FKBP-like peptidyl-prolyl cis-trans isomerase family protein (AT1G73655), mRNA	2
NM_121513.5	Arabidopsis thaliana voltage dependent anion channel 3 (VDAC3), mRNA	2
NM_100267.4	Arabidopsis thaliana FASCICLIN-like arabinoogalactan 9 (FLA9), mRNA	2
NM_102111.4	Arabidopsis thaliana myb domain protein 3 (MYB3), mRNA	1
NM_102762.5	Arabidopsis thaliana translation elongation factor EF1B/ribosomal protein S6 family protein (eEF-1Bb1), mRNA	1
NM_101873.3	Arabidopsis thaliana PAM domain (PCI/PINT associated module) protein (EMB2719), mRNA	1
NM_117647.3	Arabidopsis thaliana Deoxyxylulose-5-phosphate synthase (CLA1), mRNA	1
NM_116495.4	Arabidopsis thaliana Aldolase-type TIM barrel family protein (AT4G02610), mRNA	1
NM_130265.4	Arabidopsis thaliana phytochrome interacting factor 3-like 1 (PIL1), mRNA	1
NM_119174.2	Arabidopsis thaliana xyloglucan endotransglucosylase/hydrolase 18 (XTH18), mRNA	1
NM_123038.3	Arabidopsis thaliana transmembrane protein (AT5G36800), mRNA	1
NM_105934.2	Arabidopsis thaliana translocase inner membrane subunit 23-2 (TIM23-2), mRNA	1
NM_106294.3	Arabidopsis thaliana HSP20-like chaperones superfamily protein (AT1G76440), mRNA	1
NM_121676.4	Arabidopsis thaliana dehydroascorbate reductase 1 (DHAR3), mRNA	1
NM_110994.4	Arabidopsis thaliana voltage dependent anion channel 1 (VDAC1), mRNA	1
NM_129536.5	Arabidopsis thaliana ribonuclease 2 (RNS2), mRNA	1
NM_105527.5	Arabidopsis thaliana alpha-xylosidase 1 (XYL1), mRNA	1
NM_115784.3	Arabidopsis thaliana pirin (PRN), mRNA	1
NM_117387.3	Arabidopsis thaliana NAD(P)-binding Rossmann-fold superfamily protein (AT4G13180), mRNA	1
NM_106458.4	Arabidopsis thaliana trehalose-6-phosphate phosphatase (TPPB), mRNA	1
NM_129675.5	Arabidopsis thaliana Calcium-binding EF hand family protein (TCH3), mRNA	1
NM_105222.3	Arabidopsis thaliana PEBP (phosphatidylethanolamine-binding protein) family protein (FT), mRNA	1
NM_127293.3	Arabidopsis thaliana associated protein 19 (AP19), mRNA	1
NM_125836.4	Arabidopsis thaliana CHCH domain protein (AT5G64400), mRNA	1
NM_112282.3	Arabidopsis thaliana Subtilase family protein (AT3G14240), mRNA	1
NM_127224.6	Arabidopsis thaliana myb domain protein 7 (MYB7), mRNA	1
NM_112508.5	Arabidopsis thaliana GDSL-like Lipase/Acylhydrolase superfamily protein (AT3G16370), mRNA	1
NM_112085.4	Arabidopsis thaliana basic chitinase (HCHIB), partial mRNA	1
NM_113432.2	Arabidopsis thaliana Major facilitator superfamily protein (AT3G25260), mRNA	1
NM_120334.3	Arabidopsis thaliana histone H2A 12 (HTA12), mRNA	1
NM_180470.2	Arabidopsis thaliana loricrin-like protein (AT5G09670), mRNA	1

NM_115119.4	Arabidopsis thaliana ubiquitin extension protein 1 (UBQ1), mRNA	1
NM_102581.4	Arabidopsis thaliana hypothetical protein (AT1G28150), mRNA	1
NM_203202.3	Arabidopsis thaliana haloacid dehalogenase-like hydrolase family protein (AT5G53850), mRNA	1
NM_117299.7	Arabidopsis thaliana Copper amine oxidase family protein (AT4G12290), mRNA	1
NM_001341084.1	Arabidopsis thaliana proline-rich family protein (AT4G16140), mRNA	1
NM_112897.4	Arabidopsis thaliana ubiquitin-conjugating enzyme19 (UBC19), mRNA	1
NP_191096.1	3-oxo-5-alpha-steroid 4-dehydrogenase family protein [Arabidopsis thaliana]	1
NP_178443.1	nodulin-related protein 1 [Arabidopsis thaliana]	1
NP_193465.1	tonoplast intrinsic protein 2;2 [Arabidopsis thaliana]	1
NP_567494.1	transmembrane protein [Arabidopsis thaliana]	1
NP_194922.1	cytochrome P450, family 82, subfamily C, polypeptide 4 [Arabidopsis thaliana]	1
NP_001330199.1	inosine-uridine preferring nucleoside hydrolase family protein [Arabidopsis thaliana]	1
NP_563829.1	ERD (early response to dehydration) six-like 1 [Arabidopsis thaliana]	1
NP_564616.1	Peroxisomal membrane 22 kDa (Mpv17/PMP22) family protein [Arabidopsis thaliana]	1
AAA92800.1	chloroplast omega-6 fatty acid desaturase [Arabidopsis thaliana]	1
CAA0395949.1	unnamed protein product [Arabidopsis thaliana]	1
CAD5333982.1	Toll-Interleukin-Resistance (TIR) domain family protein [Arabidopsis thaliana]	1
NP_001331471.1	Duplicated homeodomain-like superfamily protein [Arabidopsis thaliana]	1
NP_001190920.1	plasma membrane intrinsic protein 3 [Arabidopsis thaliana]	1
NP_566697.1	Receptor-like protein kinase-related family protein [Arabidopsis thaliana]	1
NP_566527.1	phosphatidic acid phosphatase-related / PAP2-like protein [Arabidopsis thaliana]	1
CAA70953.1	hypothetical protein [Arabidopsis thaliana]	1
NP_175633.1	photosystem I subunit H2 [Arabidopsis thaliana]	1
NP_192696.3	SNARE associated Golgi protein family [Arabidopsis thaliana]	1
NP_199215.1	HAD superfamily, subfamily IIIB acid phosphatase [Arabidopsis thaliana]	1
BAB02684.1	peptide/amino acid transporter-like protein [Arabidopsis thaliana]	1
NP_199514.1	ATPase, F1 complex, delta/epsilon subunit [Arabidopsis thaliana]	1
NP_001330235.1	carbonic anhydrase 2 [Arabidopsis thaliana]	1
NP_565206.1	Ca(2)-dependent phospholipid-binding protein (Copine) family [Arabidopsis thaliana]	1
NP_187246.1	RmlC-like cupins superfamily protein [Arabidopsis thaliana]	1
NP_191469.3	glucan synthase-like 11 [Arabidopsis thaliana]	1
NP_186817.1	sieve element occlusion amino-terminus protein [Arabidopsis thaliana]	1
NP_568904.1	lipid transfer protein 4 [Arabidopsis thaliana]	1
NP_200780.1	heat shock protein 18.2 [Arabidopsis thaliana]	1
NP_174010.1	Ribosomal protein L34e superfamily protein [Arabidopsis thaliana]	1
NP_174116.1	glycosylphosphatidylinositol-anchored lipid protein transfer 1 [Arabidopsis thaliana]	1
NP_565178.1	glutathione S-transferase TAU 19 [Arabidopsis thaliana]	1
NP_177726.1	PAM domain (PCI/PINT associated module) protein [Arabidopsis thaliana]	1
NM_111175.3	Arabidopsis thaliana cellulose synthase-like D3 (CSLD3), mRNA	1
NM_119728.2	Arabidopsis thaliana phosphoserine aminotransferase (PSAT), mRNA	1
NM_115941.4	Arabidopsis thaliana Ribosomal protein S13/S15 (AT3G60770), mRNA	1
NM_001337948.1	Arabidopsis thaliana aluminum activated malate transporter family protein (AT3G11680), mRNA	1
NM_127871.5	Arabidopsis thaliana Protein kinase superfamily protein (AT2G23070), mRNA	1
NP_051114.1	NADH dehydrogenase subunit 1 [Arabidopsis thaliana]	1

NM_122036.5	Arabidopsis thaliana Ribosomal protein S8e family protein (AT5G20290), mRNA	1
NM_128939.4	Arabidopsis thaliana Ribosomal protein S5 family protein (EMB3113), mRNA	1
NM_001160888.2	Arabidopsis thaliana transcription factor (AT1G23965), mRNA	1
NM_112455.4	Arabidopsis thaliana fatty acid desaturase 5 (FAD5), mRNA	1
NM_105689.4	Arabidopsis thaliana CYCLIN D1;1 (CYCD1;1), mRNA	1
NM_112107.3	Arabidopsis thaliana DNA glycosylase superfamily protein (AT3G12710), mRNA	1
NM_001084291.2	Arabidopsis thaliana Class I peptide chain release factor (AT1G62850), mRNA	1
NM_101352.3	Arabidopsis thaliana DYNAMIN-like 1C (DL1C), mRNA	1
NM_201662.3	Arabidopsis thaliana hypothetical protein (DUF789) (AT2G01260), mRNA	1
NM_119173.4	Arabidopsis thaliana xyloglucan endotransglucosylase/hydrolase 24 (XTH24), mRNA	1
NM_119799.4	Arabidopsis thaliana beta-galactosidase 3 (BGAL3), mRNA	1
NM_119446.3	Arabidopsis thaliana glycine-rich protein (AT4G32920), mRNA	1
NM_112434.3	Arabidopsis thaliana Rubredoxin-like superfamily protein (AT3G15640), mRNA	1
NM_112675.5	Arabidopsis thaliana Galactose mutarotase-like superfamily protein (AT3G17940), mRNA	1
NM_105882.3	Arabidopsis thaliana Cupredoxin superfamily protein (AT1G72230), mRNA	1
NM_122610.3	Arabidopsis thaliana Zim17-type zinc finger protein (AT5G27280), mRNA	1
NM_118265.5	Arabidopsis thaliana PapD-like superfamily protein (AT4G21450), mRNA	1
NM_128109.3	Arabidopsis thaliana Drug/metabolite transporter superfamily protein (AT2G25520), mRNA	1
NM_104433.5	Arabidopsis thaliana SKU5 similar 12 (sks12), mRNA	1
NM_128557.4	Arabidopsis thaliana Protein phosphatase 2C family protein (AT2G30020), mRNA	1
NM_121942.5	Arabidopsis thaliana rhodanese-like domain-containing protein / PPIC-type PPIASE domain-containing protein (AT5G19370), mRNA	1
NM_117882.3	Arabidopsis thaliana syntaxin of plants 23 (SYP23), mRNA	1
NM_116156.5	Arabidopsis thaliana Peptide chain release factor 1 (APG3), mRNA	1
NM_120999.3	Arabidopsis thaliana Octicosapeptide/Phox/Bem1p family protein (AT5G09620), mRNA	1
NM_106467.4	Arabidopsis thaliana E3 ubiquitin-protein ligase (AT1G78170), mRNA	1
NM_116316.2	Arabidopsis thaliana ATPase, F1 complex, OSCP/delta subunit protein (AT4G00895), mRNA	1

Supplementary Table 2. List of primers used in the study

Primer name	Sequence 5'-3'	Purpose
For gene knockout		
E1-Hind1-L	AAGCTTCGGATTGATACACGAGTGACATT	*SsPINE1* 5' flanking sequence
E1-Sal1-R	GTCGAC CCATCAGCTGTTCATCAGCTGACTC	*SsPINE1* 5' flanking sequence
E1-Xba1-L	TCTAGA ACTGCATTCGGAAGAAGAAGGTTTG	*SsPINE1* 3' flanking sequence
E1-Knp1-R	GGTACC TGCTGAGAAAATTGCGGCACAACCT	*SsPINE1* 3' flanking sequence
Sac1CE1-L	GAGCTCATGAAGCTCAGTCAACCACTAAGCAT	*SsPINE1* complement/RT-PCR
Kpn1CE1-R	GGTACCCTTGTACAGCTCGTCCATGCCGA	*SsPINE1* complement/RT-PCR
E1YZ-L-1	GGGATTCCCACAGGCCAAACC	Outside 5' PINE1 construct for confirmation
PG1-Hind-L	AAGCTTCTTCCAATACCTTCTTTTCTTTC	*SsPG1* 5' flanking sequence
PG1-Sal-R	GTCGACCAGAGGCAGAGTTGATGGAGA	*SsPG1* 5' flanking sequence
PG1-Xba1-L	TCTAGACGTTTACGTTCTCTGCGCCAAGTGC	*SsPG1* 3' flanking sequence
PG1-Kpn1-R	GGTACCCAAAATCATATGCACCTCAACAACA	*SsPG1* 3' flanking sequence
PG1-Sac1	GAGCTC ATGGTTGAGATTCTTTCCTCGGCCT	*SsPG1* complement/RT-PCR
PG1-SMA1	CCCGGGACACTTGACACCAGATGGGAGACCA	*SsPG1* complement/RT-PCR
PGYZ-L	TTTGATATATGTGATATGATCAC	Outside 5'PG1 construct for confirmation
YG-F	GATGTAGGAGGGCGTGGATATGTCCT	Hyg Split 3' construct
HY-R	GTATTGACCGATTCCTTGCGGTCCGAA	Hyg Split 5' construct
YZPG1-L	ACTCCCTCGGACACAACACT	*SsPG1* RT-PCR
YZPG1-R	TTGGTAGCGGAGGAAGAGACGG	*SsPG1* RT-PCR
YZ-E1-L	GGGATTCCCACAGGCCAAACC	*SsPINE1* PCR
YZ-E1-R	TTCACGAACAATCTCACCCC	*SsPINE1* PCR
BoE15-L	CAGTTTGGTTTCCAAAGTACAATG	*BcPINE1* 5' flanking sequence
BoE15 -R	CCTTTTTTTTTCAGGAATTATTCTCACAGTGAGGAAACAGATGAAAGCAGGG	*BcPINE1* 5' flanking sequence
BoE13-L	gatctagatgcattcgcgaggtaccgagctAATATCATGGCAGGCTTACAACG	*BcPINE1* 3' flanking sequence
BoE13-R	CCTTCCTCAAGGTTACCACCAAAG	*BcPINE1* 3' flanking sequence
YE1F	CATGGAGGCCGAATTCGTTCCTCAAGACGATCAATGC	Yeast 2-hybrid screening
YE2R	GCAGGTCGACGGATCCTCACTTGCAAACGCTGTAGT	Yeast 2-hybrid screening
SsPINE1$^{\Delta SP}$-NED-L	CATATGATGGTTCCTCAAGACGATGC	Yeast two-hybrid
SsPINE1 -BAMH1-R	GGATCCCTTGCAAACGCTGTAGTCCG	Yeast two-hybrid
AtPGIP1-NED-L	CATATGATGGATAAGACAGCGACATTGTGTC	Yeast two-hybrid
AtPGIP1-BAMH1-R	GGATCCCTTGCAAATTTCAAGAGGAGCACCA	Yeast two-hybrid
AtPGIP2-NED-L	CATATGATGGATAAGACAATGACACTGTTC	Yeast two-hybrid

AtPGIP2-BAMH1-R	GGATCCCTTGCAACTAGGAAGAGGTGCACC	Yeast two-hybrid
AtPGIP1-Sal1-L	GTCGACATGGATAAGACAGCGACATTGTGTC	BiFC/Co-IP
AtPGIP1-Sam1-R	CCCGGGCTTGCAAATTTCAAGAGGAGCACCA	BiFC/Co-IP
SsPINE1-Sal1-L	GTCGACATGAAGCTCAGTCAACCACTAAGCAT	BiFC/Co-IP
SsPINE1$^{\Delta SP}$-Sal1-L	GTCGACATGGTTCCTCAAGACGATGC	BiFC/Co-IP
SsPINE1-Sam1-R	CCCGGGCTTGCAAACGCTGTAGTCCGGT	BiFC/Co-IP
SsPG1-Sal-L	GTCGACATGGTTGAGATTCTTTCCTCGGCCT	BiFC/Co-IP
SsPG1-Sma1-R	CCCGGGACACTTGACACCAGATGGGAGACCA	BiFC/Co-IP
GFP-Sma1-L	CCCGGGATGGTGAGCAAGGGCGAGGAGCTGT	Co-IP
GFP-Sac1-R	GAGCTCCTTGTACAGCTCGTCCATGCCGAGA	Co-IP
SsPG1-L	TTGTCTTCGAGGGTACTACCAC	RT-qPCR
SsPG1-R	CTTTCCACCGTTGGTACCCTT	RT-qPCR
SsPG3-L	ATGCTGATATTGCCTCCGTTG	RT-qPCR
SsPG3-R	CCATCCCAGTATGCAGGTCCA	RT-qPCR
SsPG5-L	TACGGTATCATCGTCAACCAAGC	RT-qPCR
SsPG5-R	TCCATGTCCAGTTAGTGCAAG	RT-qPCR
SsPG6-L	TCTTAGCCCTCACTTTAGCAG	RT-qPCR
SsPG6-R	AGTGATTTTAGTACCGGCCTT	RT-qPCR
SsPINE1-L	ATTCTCGCAGCATCAACTGTC	RT-qPCR
SsPINE1-R	AATCCATCACAACAGGTGCCAA	RT-qPCR
Tub-F	TTGGATTTGCTCCTTTGACCAG	RT-qPCR
Tub-R	AGCGGCCATCATGTTCTTAGG	RT-qPCR
AtPGIP1-L	CCAAACTCAAGAACCTCCGAA	RT-qPCR
AtPGIP1-R	TTCCTGCTAAGTTCAAGAGCC	RT-qPCR
UBQ-F	ACACCAAGCCGAAGAAGA	RT-qPCR
UBQ-R	TCCACAGGTTGCGTTAGG	RT-qPCR
AT5G64120-L	CTCGTGACACAGTCATTCTCACTC	RT-qPCR
AT5G64120-R	GTTGTTGAACGGCAACGGAGTC	RT-qPCR
AT5G48180-L	TGTTTCCGGCGGTTTCTTACGG	RT-qPCR
AT5G48180-R	TAAGCTCATGCGGCTCTTCCTC	RT-qPCR
AT1G77510-L	TTGAAAGCCCTGCCACTAAGGC	RT-qPCR
AT1G77510-R	TCTGCATCACCAACAAGGAAGGC	RT-qPCR
AT2G45220-L	AGCAAGGCGTTTACAGTGAGAACC	RT-qPCR
AT2G45220-R	CCGCGACAGTAGCTGAGTTAAAGG	RT-qPCR
AT1G26390-L	ACGCCACAATGAGTAGCCTGAG	RT-qPCR
AT1G26390-R	TCACCACTCGGATTGCTTCCAAC	RT-qPCR
AT4G38970-L	CTCTTCCGTCAGCCTTCTTC	RT-qPCR

AT4G38970-R	GCAGAGACCAGCAAAGTCCGG	RT-qPCR
AT1G12440-L	AGCAAAACGACAGCACAAGC	RT-qPCR
AT1G12440-R	GGTTGAGTTTTGGGTTTATTAGGGT	RT-qPCR
AT4G09010-L	GCTGCTGACTTGAATCAACGTAGAC	RT-qPCR
AT4G09010-R	GAAAGTCCCTCATTCTCTGCTC	RT-qPCR
AT3G54050-L	TGGCATCATCATCCCAGCCTTCAAT	RT-qPCR
AT3G54050-R	GCATCTATCTCTCCTTTCATCTCTT	RT-qPCR
AT5G52470-L	TGGTGGAGGATTCAGAGGTG	RT-qPCR
AT5G52470-R	AATCACTTTGCTTCCTCCTTTC	RT-qPCR
YEST-E1ECOR1-L	GAATTC ATG GTTCCTCAAGACGATCAATGC	Yeast Expression
YESTE1GFPNOT-R	GCGGCCGC TTAATGATGATGGTGATGATG CTTGTACAGCTCGT CCATGCCGA	Yeast Expression
YEST-AD1RCOR1-L	GAATTC ATG GATCTCTGTAACCAAAATGACAA	Yeast Expression
YEST-AD1NOT1-R	GCGGCCGCTTAATGATGATGGTGATGATG CTTGCAAATTTCAAGAGGAGCA	Yeast Expression
YEST-PG1ECOR1-L	GAATTC ATG CCAGCACCAGCACCAACTGCTGCAC	Yeast Expression
YEST-PG1NOT-R	GCGGCCGCTTAATGATGATGGTGATGATG ACACTTGACACCAGATGGGAGA	Yeast Expression
For biomass determination		
For *Sclerotinia SsTub1 gene*		
SsTub-F:	ACCTCCATCCAAGAACTC	
SsTub-R:	GAACTCCATCTCGTCCAT	Biomass determination
For pea *PsGAPDH*		
PEAGAPDH-L	GTCTTTGCACACAGGAACCCA	
PEAGAPDH-R	GGCACCACCCTTCAAATGAG	Biomass determination
For *Arabidopsis AtUBQ5*		
ATUBQ5-L	ACACCAAGCCGAAGAAGA	
ATUBQ5-R	TCCACAGGTTGCGTTAGG	Biomass determination
For *Botrytis cinerea Bcgpdh gene*		
Bcgapdh-q F	CGAAGAATAGCACAAACAGCTGGAC	
Bcgapdh-q R	CGTCACCTTATGCTTCTTGCTCC	Biomass determination

Supplementary Table 3. List of plasmids used in study

Plasmid name	Vector	Purpose
pCETNSE1	pCETNS	Complement and overexpression of SsPINE1 in *S. sclerotiorum*
pCETNSPG1	pCETNS	Complement and overexpression of SsPG1 in *S. sclerotiorum*
pCH-E1	pCH3300	For SsPINE1 Knockout constructs
pCH-PG1	pCH3300	For SsPG1 Knockout constructs
pCNRCM-SsPINE1	pCNRCM3	BiFC (SsPINE1-cRFP)
pCNRCM-SsPINE1$^{\Delta SP}$	pCNRCM3	BiFC (SsPINE1$^{\Delta SP}$-cRFP)
pCNRCM-SsPG1	pCNRCM3	BiFC (SsPG1-cRFP)
pCNRNF-AtPGIP1	pCNRNF3	BiFC (AtPGIP1-nRFP)
pCHNF3- AtPGIP1	pCHNF3	Co-IP and Arabidopsis transformation
pCNG-SsPINE1	pCNG	Co-IP and Arabidopsis transformation
pCNG-SsPG1	pCNG	Co-IP
pPIC- SsPINE1	pPIC9K	Expression of *SsPINE1-GFP* in *P. pastorisi*
pPIC- SsPG1	pPIC9K	Expression of *SsPG1-GFP* in *P. pastorisi*
pPIC-AtPGIP1	pPIC9K	Expression of *AtPGIP1* in *P. pastorisi*

Supplementary Table 4. Fungal strains and Arabidopsis lines used or generated in the study

Strain	Genotype	Source
Sclerotinia sclerotiorum		
WMA1 (ATCC MYA-5421)	Wild type	Xu et al., 2015
KOE1-1; -3	WMA1-ΔSsPINE1	This study
ComE1-1; -3; -4	WMA1-ΔSsPINE1-trpC promoter::SsPINE1-GFP	This study
KOPG1-1.-	WMA1-ΔSsPG1	This study
ComSsPG1-1/ComSsPG1-/ComSsPG1-1	WMA1-ΔSsPG1-trpC promoter::SsPG1-GFP	This study
OESsPINE1	WMA1-trpC promoter::SsPINE1-GFP	This study
OESsPG1	WMA1-trpC promoter::SsPG1-GFP	This study
Botrytis cinerea		
B05.10	Wild type	Zhu et al
KoBcPINE1-3	B05.10--ΔBcPINE1	This study
KoBcPINE1-6	B05.10--ΔBcPINE1	This study
Pichia pastoris		
GS115	*his4*	Invitrogen
PSsPINE1-7	GS115 pPIC-SsPINE1$^{\Delta SP}$ -GFP-His	This study
PSsPG1-2	GS115 pPIC-SsPG1$^{\Delta SP}$ -GFP-His	This study
PSsAtPGIP1-22	GS115 pPIC-AtPGIP1$^{\Delta SP}$ -GFP-His	This study
Arabidopsis thaliana		
Col-0	Wild type	
35S::SsPINE1-5/35S::SsPINE1-8	Col-0 35S promoter::SsPINE1-GFP	This study
35S::AtPGIP1-2/35S::AtPGIP1-4	Col-0 35S promoter:: SsPGIP1-GFP	This study
