# Supplementary material for: A fungal extracellular effector inactivates plant polygalacturonase-inhibiting protein
Source: Nat Commun. 2022 Apr 25;13:2213. doi: 10.1038/s41467-022-29788-2 (PMC9038911; doi:10.1038/s41467-022-29788-2)
Supplement: Supplementary file 3 — Reporting Summary [file 41467_2022_29788_MOESM3_ESM.pdf]

nature portfolio

Corresponding author(s): Weidong Chen

Last updated by author(s): Feb 28, 2022

Reporting Summary

Nature Portfolio wishes to improve the reproducibility of the work that we publish. This form provides structure for consistency and transparency in reporting. For further information on Nature Portfolio policies, see our Editorial Policies and the Editorial Policy Checklist.

Statistics

For all statistical analyses, confirm that the following items are present in the figure legend, table legend, main text, or Methods section.

n/a	Confirmed	
☐	☒	The exact sample size (*n*) for each experimental group/condition, given as a discrete number and unit of measurement
☐	☒	A statement on whether measurements were taken from distinct samples or whether the same sample was measured repeatedly
☐	☒	The statistical test(s) used AND whether they are one- or two-sided<br>*Only common tests should be described solely by name; describe more complex techniques in the Methods section.*
☒	☐	A description of all covariates tested
☒	☐	A description of any assumptions or corrections, such as tests of normality and adjustment for multiple comparisons
☐	☒	A full description of the statistical parameters including central tendency (e.g. means) or other basic estimates (e.g. regression coefficient) AND variation (e.g. standard deviation) or associated estimates of uncertainty (e.g. confidence intervals)
☐	☒	For null hypothesis testing, the test statistic (e.g. *F*, *t*, *r*) with confidence intervals, effect sizes, degrees of freedom and *P* value noted<br>*Give P values as exact values whenever suitable.*
☒	☐	For Bayesian analysis, information on the choice of priors and Markov chain Monte Carlo settings
☒	☐	For hierarchical and complex designs, identification of the appropriate level for tests and full reporting of outcomes
☒	☐	Estimates of effect sizes (e.g. Cohen's *d*, Pearson's *r*), indicating how they were calculated

Our web collection on statistics for biologists contains articles on many of the points above.

Software and code

Policy information about availability of computer code

Data collection	Leica, Confocal laser scanning microscope TCS SP8X (Leica Microsystems) for fluorescence microscopy; ChemiDoc Touch Imaging System (Bio-RAD) for Western Blot, Cannon G11 camera for photographing disease assays and fungal morphology.
Data analysis	SignalP was used to identify secreted proteins and their SPs. BlastP analysis was done on the website of NCBI(http://www.ncbi.nlm.nih.gov/); SAS Views for statistical analysis; originPro8 software for graphing

For manuscripts utilizing custom algorithms or software that are central to the research but not yet described in published literature, software must be made available to editors and reviewers. We strongly encourage code deposition in a community repository (e.g. GitHub). See the Nature Portfolio guidelines for submitting code & software for further information.

Data

Policy information about availability of data

All manuscripts must include a data availability statement. This statement should provide the following information, where applicable:

- Accession codes, unique identifiers, or web links for publicly available datasets
- A description of any restrictions on data availability
- For clinical datasets or third party data, please ensure that the statement adheres to our policy

The data that support the findings of this study are available within this paper, its supplementary information files, and the source data file. Accession numbers for all proteins referred to this manuscript are given in data availability section and are accessible from NCBI (https://www.ncbi.nlm.nih.gov/).

Field-specific reporting

Please select the one below that is the best fit for your research. If you are not sure, read the appropriate sections before making your selection.

- [x] Life sciences
- [ ] Behavioural & social sciences
- [ ] Ecological, evolutionary & environmental sciences

For a reference copy of the document with all sections, see nature.com/documents/nr-reporting-summary-flat.pdf

Life sciences study design

All studies must disclose on these points even when the disclosure is negative.

Sample size	No statistic methods were used to pre-determine sample size. For fungal growth assays we had three biological replicates. The variations were small between replicates and differences were significant between groups. Experiments were performed three times to confirm results.. Sample sizes in the plant infection experiments were based on previous studies in Sclerotinia sclerotiorum, where usually three biological replicates have provided statistical significant findings and based on prior experience. Six biological replicates were used in infection of Arabidopsis.
Data exclusions	No data were excluded
Replication	All experiments were subject three independent biological replications unless otherwise stated. Number of repeats is given in the figure legend. Technical replications were also carried out as stated in the text.
Randomization	All experimental observations were carried out without pre-selection of groups. Leaves of pea and Arabidopsis plants from the same individual batches were randomly assigned to treatment groups for check, WT S. sclerotiorum and mutant strains for inoculation. No other forms of randomization was relevant to this study.
Blinding	Blind testing was not carried out in the study as it was not relevant to the experiments carried out as these were based on molecular genetic and biochemical characterizations of the SsPINE1, SsPG1 and AtPGIP1 genes, requiring knowledge of the samples to be processed.

Reporting for specific materials, systems and methods

We require information from authors about some types of materials, experimental systems and methods used in many studies. Here, indicate whether each material, system or method listed is relevant to your study. If you are not sure if a list item applies to your research, read the appropriate section before selecting a response.

Materials & experimental systems

n/a	Involved in the study	
[ ]	[x]	Antibodies
[x]	[ ]	Eukaryotic cell lines
[x]	[ ]	Palaeontology and archaeology
[x]	[ ]	Animals and other organisms
[x]	[ ]	Human research participants
[x]	[ ]	Clinical data
[x]	[ ]	Dual use research of concern

Methods

n/a	Involved in the study	
[x]	[ ]	ChIP-seq
[x]	[ ]	Flow cytometry
[x]	[ ]	MRI-based neuroimaging

Antibodies

Antibodies used	Western blot hybridization: anti-Actin antibodies (Sigma-Aldrich Cat No. A3853; 1:10,000 dilution); GFP(B-2) HRP antibodies (Santa Cruz Biotechnology Cat SC-9996 HRP; 1:400 dilution); Anti-FLAG M2 mAb (Sigma-Aldrich Cat SLRW 9109; 1:10,000 diltuion); BM Chemiluminescence Western Blotting Kit (Mouse/Rabbit), Roche Diagnostics; anti-Myc (Invetro.gen)<br>In Vitro Pulldown: Anti-GFP Nanobody Affinity Gel (Biolegend Cat 689302), Anti-Flag M2 Affinity Gel (Sigma-Aldrich Cat A2200); Anti-6X His EPITOPE TAG (RABBIT) Antibody (ROCKLAND Cat No.666-401-382S)
Validation	Validation of all primary antibodies are available on the manufacturer's websites: anti-Actin antibodies (https://www.sigmaaldrich.com/life-science/cell-biology/antibodies/antibody-products.html?TablePage=107575719); GFP(B-2) HRP antibodies (Santa Cruz Biotechnology Cat SC-9996 HRP) (https://www.scbt.com/p/gfp-antibody-b-2); Anti-FLAG M2 mAb (Sigma-Aldrich Cat SLRW 9109) (https://www.sigmaaldrich.com/catalog/product/sigma/f3165?lang=en&region=US&gclid=EAIaIQobChMIzp7Zobqa5gIVCttkCh0M-genEAAYAyAAEglvrfD_BwE); Anti-Myc antibodies (https://www.thermofisher.com/antibody/product/c-Myc-Antibody-clone-9E10-3-Monoclonal/MA5-12080); BM Chemiluminescence Western Blotting Kit, (https://www.sigmaaldrich.com/catalog/product/roche/11520709001?lang=en&region=US); Anti-GFP Nanobody Affinity Gel (Biolegend Cat 689302) (https://www.sigmaaldrich.com/catalog/search?interface=All&term=ANTI-FLAG+M2+Affinity+Agarose+Gel&N=0&mode=partialmax&focus=product&lang=en&region=US); Anti-Flag M2 Affinity Gel (Sigma-Aldrich Cat A2200) (https://www.sigmaaldrich.com/catalog/search?interface=All&term=ANTI-FLAG+M2+Affinity+Agarose

+Gel&N=0&mode=partialmax&focus=product&lang=en&region=US); https://www.rockland.com/categories/primary-antibodies/6x-his-epitope-tag-antibody-600-401-382/
